# Natural display of nuclear-encoded RNA on the cell surface and its impact on cell interaction

**DOI:** 10.1186/s13059-020-02145-6

**Published:** 2020-09-10

**Authors:** Norman Huang, Xiaochen Fan, Kathia Zaleta-Rivera, Tri C. Nguyen, Jiarong Zhou, Yingjun Luo, Jie Gao, Ronnie H. Fang, Zhangming Yan, Zhen Bouman Chen, Liangfang Zhang, Sheng Zhong

**Affiliations:** 1 Department of Bioengineering, University of California San Diego, San Diego, CA 92093 USA; 2 Department of NanoEngineering, University of California San Diego, San Diego, CA 92093 USA; 3 Department of Diabetes Complications and Metabolism, Beckman Research Institute, City of Hope, Duarte, CA 91010 USA

**Keywords:** Cell surface, Extracellular RNA, Cell membrane, Single cell, Cell-environment interaction, Monocyte, Endothelial cells

## Abstract

**Background:**

Compared to proteins, glycans, and lipids, much less is known about RNAs on the cell surface. We develop a series of technologies to test for any nuclear-encoded RNAs that are stably attached to the cell surface and exposed to the extracellular space, hereafter called membrane-associated extracellular RNAs (maxRNAs).

**Results:**

We develop a technique called Surface-seq to selectively sequence maxRNAs and validate two Surface-seq identified maxRNAs by RNA fluorescence in situ hybridization. To test for cell-type specificity of maxRNA, we use antisense oligos to hybridize to single-stranded transcripts exposed on the surface of human peripheral blood mononuclear cells (PBMCs). Combining this strategy with imaging flow cytometry, single-cell RNA sequencing, and maxRNA sequencing, we identify monocytes as the major type of maxRNA+ PBMCs and prioritize 11 candidate maxRNAs for functional tests. Extracellular application of antisense oligos of *FNDC3B* and *CTSS* transcripts inhibits monocyte adhesion to vascular endothelial cells.

**Conclusions:**

Collectively, these data highlight maxRNAs as functional components of the cell surface, suggesting an expanded role for RNA in cell-cell and cell-environment interactions.

## Introduction

The cell surface is the crucial interface between the interior and exterior of the cell. Bioactive molecules, including proteins, glycans, lipids, and their chemically modified variations, are essential for the cell surface functions, e.g., extracellular signal sensing, extracellular matrix anchoring, and antigen presentation. In contrast, the contribution of nucleic acids particularly RNAs to the cell surface functions is largely unknown.

Typically, the nuclear genome encoded RNAs (ngRNA) are not expected to be present on the surface of eukaryotic cells with intact cell membranes [1]. There have been a few cases suggesting exceptions. Ribonucleoproteins were shown to be released outside of the damaged membranes from dying cells and became autoantigens [1, 2]. Another study showed that some extracellular ngRNAs were weakly attached to the membranes of cultured eukaryotic cells but are easily washed off [3]. Thus, it was unclear whether such promiscuous cell surface RNA attachment mediates any biological functions.

Several previous studies have alluded to the existence of relatively stable RNA-cell-surface interaction and the possibility of exposing ngRNA fragments to the extracellular surface of intact cells under physiological conditions. The first membrane-bound RNAs were discovered in bacteria [4, 5], and they are non-coding RNAs transcribed from the same operon as the genes encoding transmembrane proteins. Following co-transcription, the non-coding RNA and the transmembrane protein assemble into a ribonucleoprotein that gets incorporated into the cell membrane [4, 5]. In human cells, it was shown that a few ngRNAs can bind to membrane lipids under physiological ionic conditions [6, 7]. Furthermore, atomic force microscopy data suggest that RNAs can coat the surface of artificial phospholipid membranes [8].

Here, we examined the presence of ngRNAs that are stably associated with the surface of the intact mouse and human cells and exposed to the extracellular milieu. We termed these RNAs membrane-associated extracellular RNAs (maxRNA). This definition does not include the RNAs encapsulated within any cellular or extracellular vesicles [9] or cell-free RNAs that are not stably attached to cell membranes [3]. Furthermore, we characterized the sequences, cell-type specificity, and functional attributes of maxRNAs with a focus on those that are present on human peripheral blood-derived mononuclear cells (PBMCs) as the archetype.

## Results

### Surface-seq: characterization of maxRNAs by sequencing

To inspect the possible cell surface-presentation of nuclear-encoded RNA, we developed a technology called Surface-seq. Surface-seq is based on a nanotechnology that extracts the plasma membrane from cells and tightly assembles the membrane around polymeric cores to form membrane-coated nanoparticles (MCNP) [10–12]. This technology suits our purpose because it retains the inside-outside orientation of the membrane by keeping the surface molecules on the membrane facing outwards, as validated by transmission electron microscopy [10, 13]. Moreover, the process of cell membrane purification and their stable coating onto the polymeric core ensures the rigorous removal of intracellular contents [10–12]. These validated features of MCNP enable the purification of RNAs that are stably associated with the extracellular layer of the cell membrane [10, 12], which are then used as the input of the Surface-seq library construction and sequencing.

**Fig. 1:**
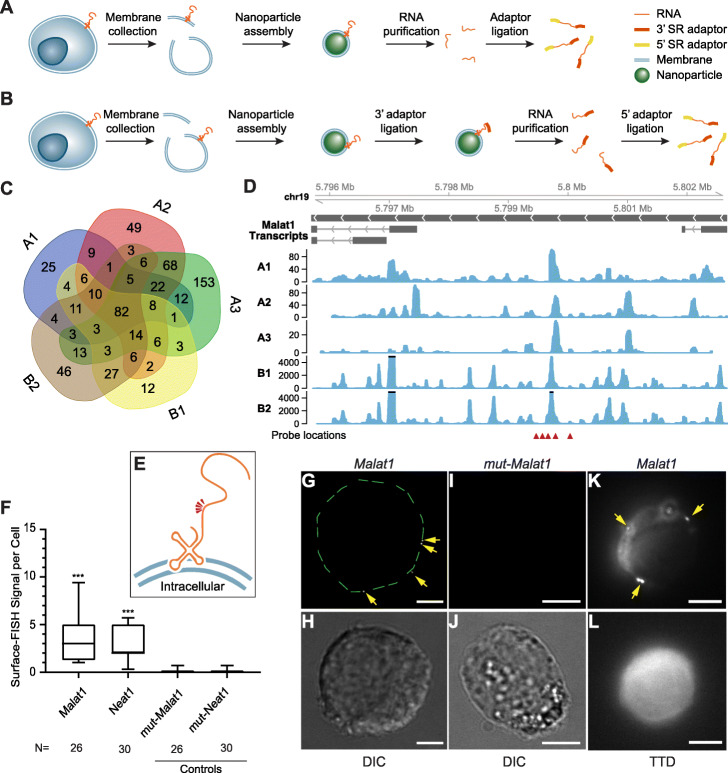
Sequencing and validation of maxRNA from a cell line. **a**, **b** The workflow of the two variations of Surface-seq. **c** A Venn diagram of the noncoding RNAs identified by the 5 Surface-seq experiments, indexed by A1, A2, A3 (based on Surface-seq variation A), and B1, B2 (based on Surface-seq variation B). **d** Read coverages from the 5 Surface-seq libraries on the *MALAT1* gene, indexed by A1, A2, A3, B1, and B2. Red arrowheads: locations of Surface-FISH probes. **e** A hypothetical model of the relative positions of Surface-FISH probes (red arrowheads) on a membrane-bound *Malat1* RNA fragment. **f** Box plots of the numbers of Surface-FISH signal foci per cell (*y*-axis) for *Malat1*, *Neat1*, and two controls (*mut-Malat1*, *mut-Neat1*) (columns). *N*: number of cells examined. **g**, **h** *Malat1* Surface-FISH (**g**) and DIC image of the same cell (**h**). The green dashed lines outline the rim of the cell. **i**, **j** Control probeset *mut-Malat1* Surface-FISH (**i**) and DIC images of the same cell (**j**). **k**, **l** *Malat1* Surface-FISH (**k**) and transmission-through-dye (TTD) image of the same cell (**l**). Arrows: *Malat1* Surface-FISH signals. The TTD image was produced by a membrane-permeable dye used in conjunction with a membrane-impermeable quencher, indicating a cell with an intact cell membrane. Scale bar = 5 μm. Probe signals were compared against corresponding controls. ***p value < 0.0001

We performed Surface-seq with EL4 cells using 2 technical variations. In variation A, after MCNPs were assembled and washed, RNAs were extracted using phenol-chloroform, quantified, and constructed into a sequencing library (Fig. 1a, Additional file 1: Fig. S1 and S2). This variation enriches for all membrane-associated RNA without differentiating the sides of the membrane. In variation B, after the MCNP assembly, the RNAs exposed on the outer surface of MCNPs (which correspond to the outer cell surface) were directly ligated to a 3′ RNA adaptor. The RNA was subsequently purified and ligated with the 5′ adaptor. Because the 3′ adaptor was selectively ligated to the outside-facing RNA, this technical variation enriches for the outside-facing membrane-associated RNA in the sequencing library (Fig. 1b).

We generated 5 Surface-seq libraries from EL4 cells, including 3 replicate libraries from technical variation A (A1, A2, A3) and 2 replicate libraries (B1, B2) from technical variation B (Additional file 1: Table S1). Our initial analysis focused on long noncoding RNAs (lncRNAs) because these have been previously associated with bacterial or mammalian cell membrane functions [4, 7]. Each sequencing library revealed 200 to 400 lncRNAs with counts per million greater than 2, and 82 of them, including *Malat1*, *Neat1*, and *Snhg20*, were shared among all 5 Surface-seq libraries (Fig. 1c, d). Taking *Malat1* as an example, the Surface-seq reads were not uniformly spread across the entire lncRNA, but enriched at specific regions, especially around the center of the transcript (Fig. 1d). To identify the outside-facing RNAs, we compared the sequencing libraries generated from Variation B (B1, B2) to those generated from Variation A (A1, A2, A3). A total of 17 lncRNAs were identified (Benjamini-Hochberg adjustment FDR < 0.05, and fold change > 2, DESeq2 [14]), including *Malat1* (the scale of the B1, B2 tracks was larger than the scale of the A1, A2, A3 tracks, Fig. 1d). These experiments identified candidate maxRNAs that appeared consistently on the outer cell membrane for further validation.

### Validation of maxRNAs by RNA-FISH on the cell surface (Surface-FISH)

To validate the localization of candidate maxRNAs, we carried out single-molecule RNA-FISH on the cell surface, which we termed Surface-FISH. This technique was adapted from our previously established protocol [15] where the cell membrane permeabilization step was skipped. We used a set of five quantum-dot-labeled oligonucleotide probes each consisting of 40 nt against the target transcript (arrows in Fig. 1d, e). We tested 2 Surface-seq prioritized lncRNAs, i.e., *Malat1* (Fig. 1f–l) and *Neat1* (Fig. 1f) in EL4 cells. To control for probe specificity, we used probes with six mutated bases at the center of the 40 nt probes designed for testing *Malat1* (*mut-Malat1* control) and *Neat1* (*mut-Neat1* control), respectively (Additional file 1: Table S3). We examined 20 to 30 single cells for each probe-set (Fig. 1f). Nearly all cells treated with *Malat1* and *Neat1* probes exhibited Surface-FISH signals, ranging from 1 to 10 signal foci per cell, whereas most cells treated with the control probes exhibited no signal (median = 0) (*p* values < 0.0001, Wilcoxon rank tests) (Fig. 1g–j).

To confirm that the Surface-FISH signals are not a result of RNA leakage from damaged cell membranes, we combined *Malat1* Surface-FISH with a transmission-through-dye (TTD) microscopic analysis, where only live cells with intact membranes are fluorescently labeled [16–18] (Additional file 1: Fig. S3). *Malat1* FISH signals appeared on cells with perfectly intact membranes (Fig. 1k), as indicated by TTD staining of the same cell (Fig. 1l). Together, observations made using various techniques suggest the presence of specific nuclear-encoded transcripts on the surface of intact live cells.

### Visualization of maxRNA from primary PBMCs as a test for cell-type specificity

Based on the concept of guilt-by-association [19–21], cell-type specificity of maxRNA presentation may suggest the relevance of maxRNA to the functions of the presenting cells. To evaluate this association, we tested whether maxRNAs are present in primary human cells under physiological conditions and whether their presence is cell-type specific. We chose primary PBMCs for these tests, considering their heterogeneity and frequent interactions among each other and with other cell types. We collected 120,000 PBMCs from each of the 4 human subjects and split each donor’s cells into 4 aliquots, each with 30,000 cells. The 4 aliquots of PBMCs per donor were used for 1 test and 3 control experiments, as described below.

**Fig. 2:**
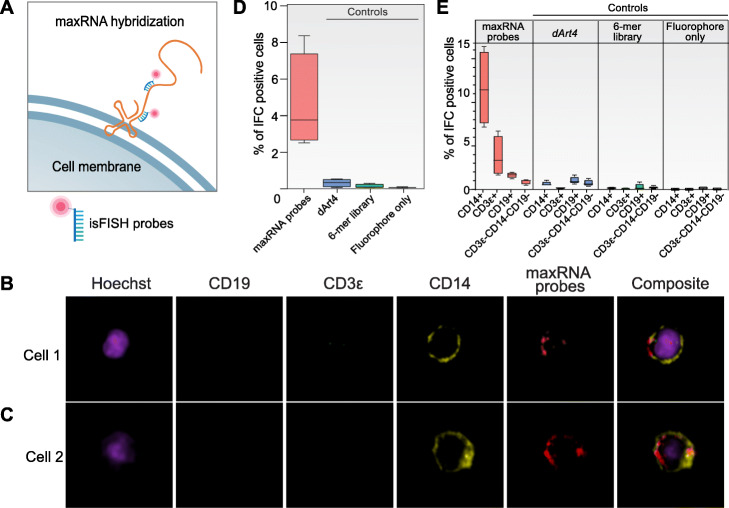
Imaging flow cytometry (IFC) analysis of maxRNAs co-localization with immune cell surface markers in human PBMCs. **a** A schematic view of the extracellular hybridization of the fluorophore (red dot) labeled probes (isFISH probes) to a hypothetical maxRNA. **b**, **c** IFC images of two representative PBMC cells (rows) in 5 channels (columns), including DAPI, CD19, CD3ε, CD14, and maxRNA probes, as well as merged images of all channels (Composite). **d**, **e** The proportions of PBMCs that exhibit IFC signals (*y*-axis) when assayed with maxRNA probes and 3 types of controls (*dArt4* probe, 6-mer library, and fluorophore only). Each box plot was derived from 4 independent biological replicates. **d** Total IFC positive cells PBMCs for each probe treatment. **e** CD14+, CD3ε+, CD19+, and CD3ε−CD14−CD19− PBMCs are separately plotted

In the test experiment, we probed for putative maxRNAs on PBMCs by hybridization with a randomized library of fluorescence-labeled oligonucleotides of 20 nt (maxRNA probes). Hereafter we will refer to this technique as in situ surface FISH (isFISH) (Fig. 2a). After probe incubation and washes, we subjected PBMCs to an imaging flow cytometry (IFC) analysis of 6 channels, which detects brightfield, live/dead, cell nuclei (Hoechst), maxRNA, CD14 (a monocyte marker), CD3ε (a T cell marker), and CD19 (a B cell marker) (Fig. 2b, c, Additional file 1: Fig. S4). To evaluate any possible fluorophore internalization or non-specific membrane attachment, we carried out 3 control experiments. We replaced the 20-mer probes respectively with one of the following: (1) a randomized probe library of 6 nt oligonucleotides (6-mer library control), (2) a 20 nt probe against the drosophila *Art4* RNA (*dArt4* control), and (3) the fluorophore without conjugating any oligonucleotides (fluorophore only control).

On average, 4.8% of total PBMCs exhibited isFISH signals, which is at least 27-fold more than any of the control groups (Fig. 2d) (*p* value < 0.005, Kruskal-Wallis test). At the stereotypical cell type level, on average more than 10% of CD14+ cells and approximately 3% of CD3ε+ cells exhibited isFISH signals (*p* value < 0.005, *t* test), whereas less than 2% of CD19+ and CD3ε−CD14−CD19− cells exhibited isFISH signals (Fig. 2e). These data support the presence and cell-type specificity of maxRNA in primary human PBMCs, thereby accumulating evidence towards guilt-by-association [19–21], i.e., the relevance of maxRNA to the functions of the presenting cells.

### Single-cell transcriptome analysis of maxRNA-presenting cells: additional evidence for cell-type specificity

To provide further evidence for the cell-type specific maxRNA presentation, we characterized the maxRNA presenting cells by combining isFISH and fluorescence-activated cell sorting (FACS) with single-cell RNA sequencing (scRNA-seq). Specifically, after isFISH labeling, we performed FACS on PBMCs and obtained 2 cell populations, i.e., isFISH+ and isFISH−. We then subjected these two populations of cells scRNA-seq on the 10X Genomics platform, which yielded 2486 isFISH+ and 9043 isFISH− cells, with on average 21,059 reads per cell. The 3 control experiments (6-mer, dArt4, fluorophore only) yielded too few positive cells (Fig. 2d) to be analyzed by the 10X Genomics scRNA-seq platform.

**Fig. 3:**
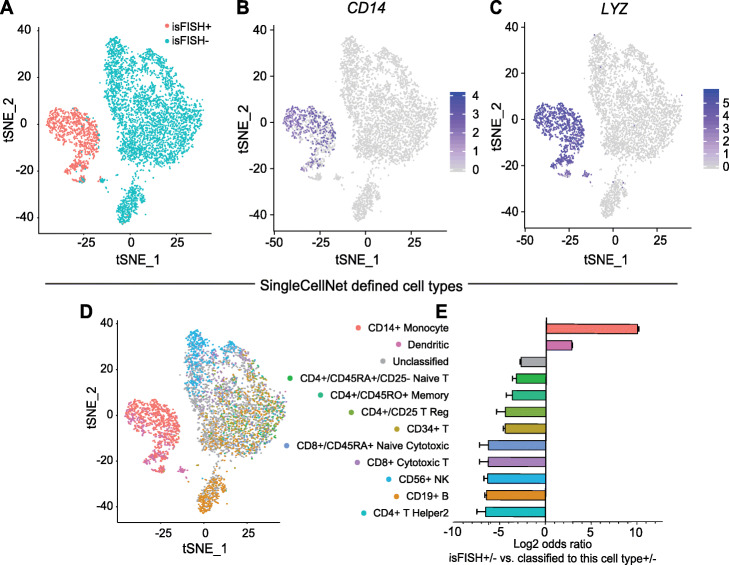
Single-cell transcriptomes of maxRNA presenting PBMCs. **a**–**c** tSNE plots of 11,233 single cells resulted from FACS sorting, including 2486 isFISH+ (pink dots) and 9043 isFISH− cells (blue dots) (**a**), with *CD14* (**b**), and *LYZ* (**c**) expression levels color-coded as indicated next to the cell type label. **d** The classification of the single cells with each color representing a pre-defined cell type. **e** Association (measured by log2 odds ratio, *x*-axis) of isFISH+ cells with each pre-defined cell type (*y*-axis). A value greater than 0 indicates enrichment, while a value smaller than 0 indicates depletion. Error bar: standard error of the log2 odds ratio

Next, we employed both unsupervised and supervised methods to query whether the isFISH+ cells are associated with any known cell types. In the unsupervised analysis, we plotted the single-cell transcriptomes on a tSNE plot (Fig. 3a). isFISH+ and isFISH− cells formed two separate clusters on the tSNE plot (blue and red dots, Fig. 3a). The single cells expressing monocyte markers *CD14* and *LYZ* were enriched in the isFISH+ cluster and were nearly absent from the isFISH− cluster (Fig. 3b, c). On the contrary, the single cells expressing T cell markers *CD3E* and *CD8A*, natural killer (NK) cell marker *NKG7*, and B cell marker *MS4A1* were enriched in the isFISH- cluster (Additional file 1: Fig. S5).

For a supervised analysis, we used the trained SingleCellNet classifier [22] that classifies each PBMC into one of its 11 pre-defined PBMC cell types (Fig. 3d). These 11 cell types were defined by training the SingleCellNet with ~ 68,000 human PBMC single-cell transcriptomes [22]. We ranked the 11 pre-defined cell types by their association with isFISH+ cells based on odds ratios (Fig. 3e). Two pre-defined cell types, namely CD14+ monocytes and dendritic cells, were enriched in isFISH+ cells (first two columns, Fig. 3e). The majority (87%) of the isFISH+ cells were classified as CD14+ monocytes, as compared to only 0.55% of isFISH− cells classified as CD14+ monocytes (odds ratio = 1143, Bonferroni adjusted *p* value < 0.001). Of the other 10 cell types, dendritic cells exhibited a modest enrichment with isFISH+ cells (odds ratio = 7.78) and the other 9 cell types were relatively depleted in isFISH+ cells (odds ratio < 1, Fig. 3e). Consistent with the unsupervised analysis, this supervised analysis suggests that the majority of maxRNA-presenting cells are monocytes. Collectively, both isFISH imaging flow cytometry (in Fig. 2) and isFISH scRNA-seq (in Fig. 3) data suggest that maxRNA are not uniformly present in all cell types, and monocytes are a major maxRNA-presenting cell type in human PBMCs.

### Antisense purification and sequencing of maxRNAs from primary human cells

**Fig. 4:**
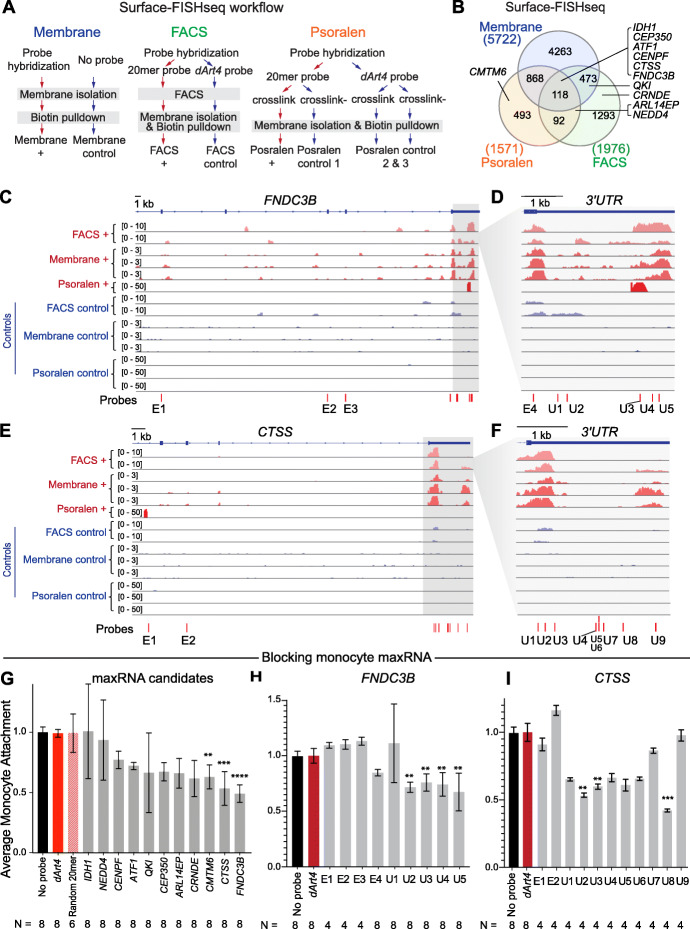
Surface-FISHseq analysis of PBMCs and functional tests of maxRNAs in primary human monocytes. **a** The Surface-FISHseq experimental workflow for the 3 technical variations. **b** A Venn diagram of maxRNAs identified by each of the 3 technical variations of Surface-FISHseq: Surface-FISHseq-membrane (blue), Surface-FISHseq-FACS (green), and Surface-FISHseq-Psoralen (orange). **c**–**f** Read distributions of Surface-FISHseq test libraries (red tracks) and control libraries (blue tracks) on (**c**) *FNDC3B* and (**e**) *CTSS*. **d**, **f** Expanded views of the 3′UTR regions of the two genes. Probes track (bottom track): locations and IDs of antisense oligonucleotides for functional tests. **g** Effects of probe incubation on the average monocyte attachment levels (normalized to the no-probe control (black), *y*-axis) of *dArt4* control probeset (*dArt4*), the randomized 20 nt control probeset (random 20-mer), and the antisense probe-sets against the 11 Surface-FISHseq identified targets (gray columns). Each probe-set is comprised of twenty-five 20 nt antisense oligonucleotide probes. Error bar: standard error of the mean. *N*: number of replicate experiments. Each condition was compared to no-probe control. **Bonferroni-adjusted *p* < 0.001, ***Bonferroni-adjusted *p* < 0.0001, ****Bonferroni-adjusted *p* < 0.00001. **h**, **i** Effects of individual antisense probes to the average monocyte attachment levels (*y*-axis) in no-probe control (black), *dArt4* control (red), and by each *FNDC3B* probe (gray columns, indexed by E1-E4, U1-U5 corresponding to locations in **d**) and each *CTSS* probe (E1, E2, U1-U9 corresponding to the locations in panel **f**). Each condition was compared to no-probe control. **Bonferroni-adjusted *p* < 0.001, ***Bonferroni-adjusted *p* < 0.0001

To interrogate the functional relevance of maxRNAs in PBMCs, we asked what are the maxRNA-producing genes in these cells. To answer this question, we developed Surface-FISHseq to sequence the isFISH-captured candidate maxRNAs. The central idea of Surface-FISHseq is to purify the cell surface RNAs through hybridization with biotin-tagged probes, and then subject the purified RNA for sequencing (Fig. 4a). Compared to the previously described BrU labeling and Surface-seq, the Surface-FISHseq enables maxRNA capture and purification from primary live cells with minimal perturbation. Additionally, it allows for a microscopic examination of probe hybridization at the cell surface before proceeding to the sequencing steps.

We reasoned that even if maxRNAs exist, their relative quantity would be significantly less than intracellular RNAs. Thus, a successful maxRNA purification procedure will have to be highly selective. To this end, we carried out 3 Surface-FISHseq experiments. The 3 experiments shared the core Surface-FISHseq experimental pipeline, and each experiment contained an additional selection step to remove non-maxRNA presenting cells or intracellular components (Fig. 4a, Additional file 1: Table S2, Fig. S6A). We did not anticipate identical results from these 3 experiments due to their differences in RNA-enrichment and membrane collection methods.

In the first experiment, we purified the cell membrane before the pulldown of the probe-RNA hybrids (Surface-FISHseq-membrane). We generated 3 Surface-FISHseq-membrane libraries from PBMC samples derived from 3 different donors (Fig. 4c, e, membrane+ tracks in red). In parallel, we generated 3 control libraries from total purified membrane RNA from these same PBMC samples (Membrane control tracks in blue, Fig. 4c, e). A comparison of Surface-FISHseq-membrane libraries against control libraries based on DEseq2 [14] resulted in 5722 RNAs at the significance level of FDR < 0.15 (blue circle, Fig. 4b), including both protein-coding and non-coding RNAs (Additional file 1: Fig. S6B).

In the second experiment, we used FACS to collect isFISH+ cells, followed by maxRNA biotin-purification and sequencing (Surface-FISHseq-FACS). We generated 2 Surface-FISHseq-FACS libraries from 2 PBMC samples of 2 donors (FACS+ tracks in red, Fig. 4c and E). Two control libraries were generated from pulldown using the *dArt4* probe, from the same 2 PBMC samples (FACS control tracks in blue, Fig. 4c, e). A comparison between the test and the control libraries based on DEseq2 [14] resulted in 1976 RNAs at the significance level of FDR < 0.15 (green circle, Fig. 4b).

In the third experiment, we used psoralen to reversibly cross-link with ultraviolet light the hybridized probes to their RNA targets. We then purified probe-bound maxRNA for sequencing (Surface-FISHseq Psoralen). Psoralen only cross-links hybridized nucleic acids and does not cross-link nucleotides with proteins, which, when combined with subsequent stringent washes, minimizes indirect interactions or promiscuously attached molecules. In total, 4 libraries were generated for this experiment, including 1 targeted maxRNA library (Psoralen+ track in red, Fig. 4c, e) and 3 control libraries. The first control library is obtained following the same procedure of the targeted maxRNA library, except omitting psoralen during the cross-linking step. The remaining 2 control libraries used a 20-nt probe against *dArt4* in place of the 20-mer oligo library and were carried out with and without psoralen cross-linking (psoralen control tracks in blue, Fig. 4c, e). A comparison of the experiment and the control libraries based on DEseq2 [14] resulted in 1571 RNAs at the significance level of FDR < 0.15 (orange circle, Fig. 4b).

Pairwise comparisons of the 3 experiments revealed significant overlaps of genes detected from each experiment (odds ratio between experiments 1 and 2 = 4.53, *p* value < 2.2e−16; odds ratio between experiments 1 and 3 = 19.65, *p* value < 2.2e−16; odds ratio between experiments 2 and 3 = 5.08, *p* value < 2.2e−16). A total of 118 maxRNA genes were identified by all 3 Surface-FISHseq experiments (intersection, Fig. 4b). Taken together, Surface-FISHseq prioritized specific maxRNAs for downstream tests of their possible relevance to cellular functions.

### Cell-cell interactions are impaired by blocking specific maxRNAs

Considering the correlation between maxRNA presentation and monocytes among all PBMC cell types, we evaluated next whether maxRNAs impact cellular functions of the monocytes. We prioritized cell-cell interactions based on the biological functions of monocytes and the requirement of surface molecules in the cell-cell interactions. As one of the major cell types in innate immune and inflammatory response, monocytes have the unique property to interact with a wide range of cell types, including platelets [23], vascular endothelial cells (ECs) [24], and smooth muscle cells [25]. Among these, monocyte-EC interaction is essential under both steady-state conditions and during inflammatory responses [24, 26]. This process, initiated by surface molecules, can be reproducibly quantified by monocyte-EC adhesion assay [24, 26]. Therefore, we chose the monocyte-EC attachment level as a functional readout of the maxRNA. We tested a total of 11 Surface-FISHseq-prioritized candidate maxRNAs, namely *IDH1*, *NEDD4*, *CENPF*, *ATF1*, *QKI*, *CEP350*, *ARL14EP*, *CRNDE*, *CMTM6*, *CTSS*, and *FNDC3B* on the monocytes isolated from PBMCs (Fig. 4b).

To perturb the maxRNA without interfering with the function of the protein encoded by the mRNA of the same gene, we used extracellular hybridization with antisense probes. Specifically, these antisense probes were designed to target regions with high Surface-FISHseq read coverage, which correspond to the exposed regions of the candidate maxRNAs (Fig. 4c–f). For each maxRNA, we designed a probe-set comprised of 25 antisense oligos (each 20 nt in length) to target the parts of the transcripts with Surface-FISHseq read coverage (test probe-sets). We incubated each probe-set with monocytes for 60 min before fluorescent-labeling and seeding them onto confluent human umbilical vein endothelial cells (HUVEC). The monocyte-EC attachment level was measured by a normalized fluorescent intensity reflecting the number of attached monocytes [27]. We also included 3 controls in the experiment. The first control was not incubated with any oligo probes (no-probe control). The second control was with a probe-set comprised of 25 antisense oligos (each 20 nt in length) against *dArt4* (*dArt4* control). The third control was a randomized 20-nt probe-set (random 20-mer control).

As expected, the two control groups did not exhibit a detectable difference in monocyte-EC attachment levels in 8 repeated experiments (the first two columns, Fig. 4g). The probe-sets against *IDH1* and *NEDD4* did not induce a detectable difference either (Fig. 4g). Although probe-sets targeting *CENPF*, *ATF1*, *QKI*, *CEP350*, *ARL14EP*, and *CRNDE* resulted in lower monocyte-EC attachment levels as compared to the controls, none of these differences reached the threshold of Bonferroni-adjusted *p* value < 0.001. Finally, the monocytes incubated with antisense probes against *CTSS*, *FNDC3B*, and *CMTM6* exhibited reduced monocyte attachment levels (Bonferroni-adjusted *p* value < 0.001, Kruskal-Wallis test) (Fig. 4g), suggesting that antisense probes against specific maxRNAs can attenuate the monocyte attachment to vascular ECs.

### Specific regions of maxRNAs modulate cell-cell interactions

To test if any specific region of a maxRNA is responsible for the reduced monocyte attachment levels, we repeated the above experiments with individual 20-nt probes. Based on Surface-FISHseq read coverage, we chose 9 probes from the *FNDC3B* probe-set for this test, including 4 probes targeting 4 exons (Exon 22, 24–26) and 5 probes targeting the 3′UTR (probe track, Fig. 4c, d). Similarly, we also included two controls, i.e., a no-probe control and a 20-nt probe against *dArt4* (*dArt4* control). As expected, the no-probe control and the *dArt4* control did not exhibit any detectable difference (black and red bars, Fig. 4h). No significant difference was detected from 5 out of the 9 tested probes (E1, E2, E3, E4, U1, Fig. 4h), suggesting that not all parts of the *FNDC3B* transcript were responsible for monocyte-EC attachment. However, each of the other 4 tested probes reduced the monocyte attachment levels as compared to the no-probe control (Bonferroni-adjusted *p* value < 0.001, Kruskal-Wallis test) (U2, U3, U4, U5, Fig. 4h). All these 4 probes targeted the 3′ tail of the 3′UTR (Fig. 4d), consistent with a reproducible Surface-FISHseq peak in the 3′ portion of the 3′UTR (pink tracks, Fig. 4d). These data suggest that not all parts of a maxRNA are equally important for their cell surface functions.

To test whether the above observation could be reproduced with another maxRNA, we repeated the experiment with 11 probes from the *CTSS* probe-set, including 1 intronic probe (E1), 1 exonic probe (E2), and 9 probes spanning the 3′UTR (bottom track, Fig. 4e, f). Neither the intronic probe (E1) nor the exon probe (E2) resulted in a significant change in the attachment levels (Fig. 4i). The U7 probe at the center and U9 probe at the 3′ end of the 3′UTR did not affect the attachment level either. However, all the other 7 probes targeting 3′UTR of CTSS show a trend towards reduction of monocyte attachment levels, with U2, U3, and U8 probes reaching the significance level of Bonferroni- adjusted *p* value < 0.001 (Kruskal-Wallis test). Taken together, monocyte-EC interactions can be modulated by extracellular hybridization of antisense oligos targeting towards specific parts of *FNDC3B* and *CTSS* transcripts. These data suggest that the exposure of maxRNAs to the extracellular milieu is required for proper cell-cell interactions.

## Discussion

### The driving question: does maxRNA exist?

Cells use surface molecules for self-presentation, intercellular recognition, and extracellular interactions. Discoveries of cell-surface receptors, glycans, and functional lipids have made fundamental impacts on biology [28–30]. Our driving question is whether there is a class of RNAs that are presented on the cell surface? And if the answer is yes, what are their functional impacts on the surface-RNA presenting cell? The difficulty in addressing this question is the lack of information on the genes, cell types, and cellular functions that may be relevant to this question.

To address these challenges, we broke the driving question into the following three parts: (1) existence: is there maxRNA in any cell type? (2) specificity: is maxRNA presentation universal to all cell types or preferential to certain cell types? (3) function: is there a functional impact of any maxRNA on its presenting cell? The first question is directly aligned with our driving question, and the answers to the second and third questions can be regarded as extension and validation of the answer to the first question.

To test the existence of maxRNA, we identified candidate maxRNAs by Surface-seq and validated specific maxRNAs with RNA-FISH on the cell surface. To test cell-type specificity, we evaluated whether maxRNA correlates with any surface protein markers or cell-type-specific transcripts. To this end, we subjected cells co-stained with maxRNA and other marker proteins to imaging flow cytometry (Fig. 2) analysis. We also subjected maxRNA presenting (maxRNA+) and maxRNA- subpopulations to single-cell transcriptome analyses. These analyses suggest that maxRNAs tend to be presented on the cell types that often involve cell-cell contacts, such as monocytes and their dendritic progeny cells.

Consistently, the identified genes producing maxRNA included those involved in cell adhesion and extracellular matrix remodeling, such as *FNDC3B* [31] and *CTSS* [32]. The functions of these genes align with the properties of monocytes in binding and transmigration through the vascular endothelium [33, 34]. For functional tests, the abovementioned indications have led us to prioritize the analyses of monocyte-EC interactions. Monocyte-EC attachment was attenuated by extracellular perturbation of certain maxRNAs. Collectively, these data indicate the existence of maxRNA.

### Survival from extracellular RNase

Due to the abundance of excreted RNase, unprotected RNA is quickly degraded in the extracellular space [35]. The remaining extracellular RNA must be protected by either encapsulation in extracellular vesicles or lipoproteins, or the steric hindrance of RNA-binding proteins or lipids [36–38]. maxRNA cannot be protected by encapsulation, leaving steric hindrance the only possible means of protection. Thus, maxRNA is expected to be the small amount of post-RNase processing RNA fragments or small RNAs, protected from further RNase digestion by the steric hindrance of the membrane proteins and lipids. Consistent with this expectation, our maxRNA sequencing data revealed RNA fragments rather than full-length transcripts (Fig. 1d and 4c–f).

### Major remaining questions: origin, stabilization, and the role of maxRNAs

Our data nominated nuclear-encoded RNA as a type of cell surface molecules in mammalian systems. These data have provoked a series of questions worthy of future investigations, especially (1) how is maxRNA transported to cell surface? (2) how is maxRNA anchored on the cell surface? and (3) how does maxRNA impact membrane functions?

### Hypotheses for maxRNA translocation to the cell surface

Like other cell surface molecules, maxRNA does not have to be produced by the surface molecule-presenting cell. There are at least three classes of pathways by which a molecule can be translocated and stabilized onto a cell’s surface. The first class includes the soluble paracrine or endocrine signaling molecules that are bound to the surface receptors on the target cells. The second class is called trogocytosis, which “is a process whereby lymphocytes (B, T and NK cells) conjugated to antigen-presenting cells extract surface molecules from these cells and express them on their own surface” [39]. The third class is the translocation of intracellular molecules onto the cell surface. In all the 3 classes, the molecule-producing cells can be dying cells [1, 39, 40]. In line with the third class of pathways, bacterial ribonucleoprotein complexes are comprised of both RNA and transmembrane proteins and the RNA is required for these complexes to localize to the cell membrane [4].

### Anchoring maxRNA on the cell surface

Plausible means of stabilization of maxRNA on the cell surface include (1) forming a ribonucleoprotein complex with a transmembrane protein, (2) covalently linked with surface glycans including glycoproteins and glycolipids, and (3) bound with membrane lipids.

### Plausible roles of maxRNAs in membrane functions

The next major question is how maxRNAs mediate membrane functions. At least 2 plausible models can be speculated. In the first model, the maxRNA contained with a ribonucleoprotein complex helps to anchor the associated protein on the cell membrane to perform its membrane-related function. If this model is correct, we should anticipate seeing all of the following: (1) a membrane-associated protein that is also an mRNA-binding protein [41], (2) the mRNA or a fragment of the mRNA of this protein is a maxRNA, and (3) this protein’s membrane-related function is disrupted if the maxRNA is perturbated. The *FNDC3B* gene in our analysis meets all these expectations in that (1) FNDC3B has recently been identified as one of the human mRNA-binding proteins [41], (2) the 3′UTR of *FNDC3B* mRNA is presented on the surface of Surface-FISH+ PBMCs (Fig. 4c, d), and (3) FNDC3B is a fibronectin protein that binds integrins to facilitate cell adhesion and cell migration [42], and perturbation of *FNDC3B* maxRNA reduced monocyte-EC adhesion (Fig. 4g, h). Thus, the *FNDC3B* maxRNA fulfills the expectations of this model.

In the second model, the maxRNA may modulate the permeability of the local membrane [43]. In line with this model, synthetic RNAs are shown to be able to bind to the cell membrane, increase membrane permeability to ions and other polar molecules [44], sustain temporary hydrate pores [44], and form passive transport channels for amino acids [45].

### Limitations of this study

We have not visualized maxRNA with sufficient resolution to differentiate the two sides of the plasma membrane. The membrane thickness was estimated to be approximately 3.7 nm [46], which is smaller than the lateral resolution (~ 20 nm) of the state of art super-resolution microscopy [47]. Electron microscopy (EM) can distinguish the lipid bilayer [48]. However, EM has a limited throughput due to “time-consuming sample preparation (and scanning)” [49]. Considering the small percentage of maxRNA presenting cells in a natural cell population, we anticipate that it will require imaging a large number of cells to obtain a set of reliable EM images of maxRNA.

We cannot completely rule out the possibility of endocytosis of the experimental reagents. However, we employed a series of methods to minimize and control for this possibility. First, all antibody labeling was carried out at 4 °C, a temperature that is generally thought to minimize cellular uptake [50]. Second, we leveraged the MCNP technology to improve the purity and control for the intra-/extracellular orientation of the collected cell membrane [12]. Third, we have confirmed a candidate maxRNA on the intact cell membrane by co-staining with Surface-FISH and a membrane-permeable dye (Fig. 1k, l, Additional file 1: Fig. S5).

Our probe incubation experiments cannot preclude endocytosis. We will discuss the technical constraints and our approach to ameliorate any possible concerns of endocytosis to the interpretation of the results.

First, our goal of these experiments is to study the cellular functions under physiological conditions. Constrained by this goal, we could not apply the methods that significantly perturb the cell’s physiology. In particular, the probe hybridization experiments could not be carried out at 4 °C because 4 °C is approximately the melting temperature of a single pyrimidine base pair. Furthermore, we could not use the endocytosis inhibitors due to their interference with membrane protein distribution [51] and the boundary morphology of the cell [52].

Second, to minimize the chances of any RNA interference (RNAi) to any intracellular mRNA due to probe internalization, our probe incubation time was limited to 1 h. Such a short incubation time without transfection was in sharp contrast to the large number of published RNAi experiments in monocytes. Monocyte RNAi protocols require overnight transfection to affect protein levels [53]. Furthermore, it is more difficult to change monocyte-EC attachment levels by RNAi. Even for the proteins that govern monocyte-EC interaction, it took 72 h of transduction and puromycin selection to observe a quantifiable change on the monocyte-EC attachment levels [27].

Third, we used 3 types of controls in the probe incubation experiments. These controls provided quantitative assessments to the degree of internalization and non-specific membrane attachment. The isFISH experiments do not require the internalized probes, if any, to hybridize to any intracellular RNA to serve as the controls.

Finally, it is difficult to expand the negative controls used in our isFISH experiments. This is because any RNA fragments including the intergenic transcripts have a chance of being maxRNA. Any oligonucleotide that is complementary to any part of the human genome may not be a bona fide negative control. Thus, our 6-mer random probe control cannot be regarded as absolutely a negative control. However, we expected these 6-mer probes to yield weaker signals due to their weaker hybridization stability than the 20-mer test probes. We hope this analysis helps to illustrate the difficulty in further expanding the collection of negative control probes.

## Conclusions

The aforementioned RNA sequencing and RNA-FISH data indicate the presence of maxRNA. There is a large variation across cell types and significant heterogeneity of the cells within each cell type in terms of their maxRNA presentation. Some maxRNA may mediate intercellular interactions. These data suggest an expanded role for RNAs in cellular life beyond previously envisioned. It can be rewarding for future studies to investigate the diversity of cell types, genes, environmental cues, and biogenesis pathways for maxRNA expression and presentation, and their contribution to cellular functions.

## Methods

### Cell lines and patient blood samples

Cell lines were obtained through ATCC and cultured with their recommending culturing protocol and media. Murine lymphoma EL4 (RRID: CVCL_0255) and human leukemia Jurkat T cells (RRID: CVCL_0367) were cultured at 37 °C, 5% CO2 in Dulbecco’s modified Eagle’s medium (DMEM) and RPMI 1640 medium (Corning), respectively. Both cell culture media were supplemented with 10% (vol/vol) heat-inactivated FBS, 2 mM glutamine, 1 mM sodium pyruvate, 1 mM MEM nonessential amino acids, and 100 U/mL each of penicillin G and streptomycin (ThermoFisher).

Leukoreduction system white blood cells (LRS-WBC) were collected from the healthy donors from the San Diego Blood Bank (UCSD IRB Project #181004XX). cultured in RPMI-1640 media (Corning) supplemented with 10% (vol/vol) heat-inactivated FBS, 2 mM glutamine, 1 mM sodium pyruvate, and 1 mM MEM nonessential amino acids (ThermoFisher).

### Surface-seq analysis of EL4 cells

#### Preparation of membrane-coated nanoparticle

EL4 cell membranes were utilized to generate membrane nanoparticles, following the previously described protocol [10]. EL4 mouse lymphoma cells were cultured in T175 flasks, collected (~ 1.2–1.5 billion total cells), and washed three times with PBS by centrifugation at 500×*g* for 5 min. Cells were then resuspended in hypotonic lysis buffer (10 mM Tris-HCl pH 7.5, 10 mM KCl, 2 mM MgCl2, 33 μL protease inhibitor cocktail (Sigma, #S8830) and 10 μL RNAsin per 10 mL of solution) and homogenized with a tight-fitting pestle for 20 passes. The homogenate is transferred into an ultracentrifuge tube and centrifuged at 20,000×*g* for 20 min. This centrifugation removes intact cells and membrane-bounded organelles, such as mitochondria, nucleus, lysosomes, Golgi, apparatus, and endoplasmic reticulum. The obtained supernatant is then transferred into another ultracentrifuge tube and centrifuged at 100,000×*g* for 35 min. The supernatant containing enzymes, ribosomes, and metabolites are discarded, and the pellet was resuspended with storage buffer (10 mM Tris-HCl pH 7.5, 1 mM EDTA, and RNasin [Promega, cat. #N2111] at 1:1000 dilution). This step is repeated one more time, and membrane protein was quantified by BCA assay.

PLGA nanoparticles were prepared by the nanoprecipitation method by adding 1 mL of PLGA (PLGA-COOH, 0.67 dL/g, 10 mg/mL in acetone) into 4-mL water, and the organic solvent was evaporated for 2 h. The membrane was diluted in storage buffer to a final protein concentration of 1 mg/mL. The PLGA nanoparticles were diluted to a final concentration of 2 mg/mL in water. Mix 1 mL of the membrane and 1 mL PLGA nanoparticle and extrude through a 400-nm polycarbonate membrane for 11 passes. The membrane-coated nanoparticles (MCNPs) are centrifuged at 21,000×*g* for 10 min. The supernatant was collected, and the pellet was resuspended in 1 mL of 10 mM Tris-HCl (pH 8.0, supplemented with RNasin at 1:1000 dilution).

#### RNA isolation from MCNPs

RNA was isolated by adding 500 μL of phenol (acid phenol: CHCl_3_ 5:1 solution (pH 4.5); Ambion cat. #AM9720) to 500 μL of nanoparticle samples (membrane concentration of 2 mg/mL and PLGA nanoparticles of 4 mg/mL) and mixed with gentle vortex for 5 s. The sample mix was incubated at 37 °C with a gentle vortex (~ 1100 rpm) for 15 min and transferred to 2 mL Phase Lock Gel Heavy tubes (Quantabio, cat.# 2302830) and centrifuged at 16,000×*g* for 5 min. The upper layer or aqueous phase was transferred to and new 2-mL Eppendorf tube and 3 M Sodium acetate (pH 5.5,1:10; Ambion) was added for RNA precipitation followed by 1.5 volume of isopropanol. Samples were incubated at − 20 °C overnight and the next day washed twice with 80% ethanol at 7500*g* for 5 min at 4 °C. RNA quantification and quality were assessed using Qubit and Bioanalyzer.

#### End repair of small RNA fragments

We carried out end repair of 5′ and 3′ ends of fragmented RNAs for library preparation. End repair was carried out using T4 Polynucleotide Kinase (NEB, cat. #MO201S) that catalyzes the transfer and exchange of Pi from the gamma position of ATP to the 5′-hydroxyl termini of the RNA fragment. It also catalyzes the removal of 3′-phosphoryl groups from 3′-phosphoryl ends. End repair was carried out by adding all the following components in a 1.5-mL Eppendorf tube: 0.5 μL of Murine RNAase inhibitor, 2 μL of 10X T4 PNK Buffer, 1 μL of T4 PNK Enzyme, 2.5 μL of Nuclease free water, and 12 μL of purified small RNAs (100 ng) in a total volume of 18 μL. The components were incubated at 37 °C for 30 min in a thermoblock followed by adding 2 μL of 10 mM ATP. Incubation continued for another 30 min. Nuclease free water was added to complete the volume to 100 μL for further purification using RNeasy MinElute Cleanup Kit (Qiagen cat. #74204). The yield and size distribution of the fragmented RNA was analyzed in the Bioanalyzer RNA Pico Chip.

#### RNA library preparation

After end repair, we continue the ligation of the 3′ and 5′ adaptors. Ligation of the 3′ SR adaptor was carried out as follows: 6 μL of input RNA (100 ng) and 1 μL of 3′SR adaptor for Illumina in a total volume of 7 μL. The mix was incubated in a pre-heated thermal cycler for 2 min at 70 °C and a transfer tube on ice. After, the following components were added: 10 μL of 2X 3′ Ligation Reaction Buffer and 3 μL of 3′ ligation mix to a final volume of 20 μL and incubated for 1 h at 25 °C in a thermal cycler. To prevent adaptor-dimer formation, hybridization of the reverse transcription primer was carried out to remove excess 3′ SR adaptors that remain free after 3′ ligation reaction by transforming single-stranded DNA adaptor into double-stranded DNA molecule, which is not substrates for ligation-mediated by T4 RNA ligase and therefore does not ligate to the 5′ SR adaptor. To hybridize the reverse transcription primer, 1 μL of the SR RT primer for Illumina was added to the 20 μL sample and bring the volume to 25.5 μL with 4.5 μL nuclease-free water. Samples were incubated in the thermal cycler as follows: 75 °C/5′, 37 °C/15′, and 25 °C/15′. Ligation of the 5′ SR adaptor was carried out as follows: 1 μL of denatured 5′ SR adaptor for Illumina, 1 μL of 10X 5′ ligation reaction buffer, and 2.5 μL of 5′ ligation mix in a final volume of 30 μL. The new mix was incubated for 1 h at 25 °C in a thermal cycler.

#### Direct 3′ adaptor ligation on MCNPs

Membrane-coated NPs generated from ~ 250 million suspension cells were centrifuged at 20,000×*g* for 20 min. The MCNP pellet was resuspended in 120 μL of the 3′ ligation reaction mix (6 μL of 3′ SR Adaptor, 60 μL of 3′ Ligation Reaction Buffer, 18 μL of 3′ Ligation Enzyme mix, 1 μL RNase inhibitor, and 35 μL 1x PBS) and incubated at 25 °C during 1 h using PCR machine. Once incubation was completed, RNA was isolated using silane magnetic beads and resuspended in 120 μL RNAse free water. RNA quality was assessed using Bioanalyzer. The isolated RNA was aliquoted in 6 PCR tubes, each with 20 μL volume. Excess of 3′ adaptor was removed by adding 1 μL of the reverse transcription primer and 4.5 μL of RNA-free water to each PCR tube containing 20 μL of RNA and incubated using the following program: 75 °C/5′, 37 °C/15′, and 25 °C/15′. The RT primer will hybridize to an unligated 3′ adaptor and will prevent the formation of adaptor-dimer formation. The following steps—5′ adaptor ligation, reverse transcription, and library amplification—were performed using standard library preparation procedures (NEB cat. # E7300L). DNA enrichment was optimized by increasing the RNA input and the number of PCR cycles. Library size distribution was determined using Bioanalyzer and library size selection with Pippin HT.

#### Computational analysis of Surface-seq data

Adapter sequences and low-quality reads were trimmed from the raw sequence data with the Trimmomatic package [54]. Sequence alignment to the mouse genome (GRCm38/mm10) was performed using STAR (v2.7.3a) [55]. featureCounts (v1.6.4) [56] was used with GENCODE mouse gene annotation (GRCm38, release M24) to summarize gene read counts. DESeq2 [14] was used for differential analysis between the libraries from two technical variations. The normalized read counts generated by DEseq2 were used to calculate the counts per million (CPM) of each gene.

### Validation of maxRNA using Surface-FISH

#### Probe design

Oligonucleotide probes were designed and synthesized by IDT to hybridize to maxRNA targets *Malat1* and *Neat1*. Negative control probes were designed by replacing the middle 6 bases with random N bases. These probes were 35–40 nt in length, with similar GC contents and melting temperatures.

#### Conjugation of quantum dots to oligonucleotide probes

Oligos were modified at the 5′ end with a primary amino group and a spacer of 30 carbons to minimize the steric hindrance of probe-RNA hybridization. The carboxyl modified quantum dots 605/705 (Invitrogen, cat. # Q22001MP) were conjugated to the amino group on the probes through EDC (carbodiimide) crosslinking reaction. The probes were subsequently purified with 0.2-μm membrane filtration (Sartorius cat. #VS0172) and 100,000 (Sartorius cat. #VS0141) molecular weight cut-off (MWCO). The retentate of the 100,000 MWCO was subjected with dynabeads MyOne SILANE purification to remove any remaining unconjugated probes. A subsequent 0.2-μm membrane filtration was used to remove any final aggregates.

#### Hybridization of suspension cell lines

Probe hybridization in fixed EL4 cells was carried out as previously described [15] without the membrane permeabilization step. Briefly, cells were fixed with 4% PFA, and no permeation was performed. Cells are then added to the previously described hybridization solution with quantum-dot-labeled probes. Cells are then incubated at 37 °C overnight. Cells were subsequently washed twice in wash buffer (50% formamide, 2x SSC) and resuspended in 1x PBS for imaging.

#### Imaging and analysis

Cells were imaged in 1x PBS through wide-field fluorescence imaging using an Olympus IX83 inverted microscope at × 60 oil immersion objective (NA = 1.4). Image processing was carried out as previously described [57]. Briefly, single transcripts were detected using an automated thresholding algorithm that searches windows of possible cutoff thresholds and selects thresholds where counts do not change within a range.

### Characterization of PBMCs by isFISH

#### PBMC isolation

Ten milliliters of the LRS-WBC samples obtained from the San Diego Blood Bank was transferred to CPT vacutainer tubes (BD cat. #362753). Blood samples were mixed by inverting the tube 8–10 times immediately before centrifugation. Blood samples were centrifuged at room temperature for 25 min at 1800×*g*. In the tissue culture hood, open a spun CPT tube and carefully discard the serum. Gently pipet up and down around the top and sides of the gel plug to dislodge and PBMCs that are stuck to it without touching the plug. Transfer to a new labeled 15-mL falcon tube and centrifuge at 400×*g* for 5 min. Wash PBMCs twice with PBS or cell media and culture cells for 30 min in suspension flasks before labeling.

#### Antisense library and control probe design

Random 20-mer libraries were generated with 20 random N bases with Alexafluor 647 conjugated to the 3′ end from IDT. Random 6-mer libraries were generated with 6 random N bases with Alexafluor 647 conjugated to the 3′ end from IDT. *dArt4* probe /5Alex647N/TTAATCATAATCGTATTGGG was synthesized from IDT with Alexafluor 647 conjugated to the 3′ end.

#### Antisense probe hybridization

Oligo libraries were denatured and linearized at 70 °C for 5 min and kept at 37 °C before hybridization. Cells were incubated with probes (10 nM final concentration) at 1 million cells/mL cell media for 1 h at 37 °C. Cells were then washed twice with 1x PBS, stained with a recommended concentration of antibodies at 4C for cell surface markers CD14 (monocytes), CD3 (T cells), CD19 (B cells), live/dead fixable yellow (BioLegend, cat. #301806 [RRID:AB_314188], 300406 [RRID:AB_314059], 302230 [RRID:AB_2073119] and 423104) and nucBlue live cell stain (Thermo Fisher cat. #R37605). For fluorophore only control Alexafluor 647 Isotype control was used (Biolegend cat. #400130). Cells were then washed twice with 1x PBS to remove excess.

#### Imaging and analysis

For imaging with microscopy, cells were imaged in 1x PBS through Widefield fluorescence imaging using an Olympus IX83 inverted microscope at × 60 oil immersion objective (NA = 1.4). For imaging with imaging flow cytometry, cells were imaged using the Amnis ImageStreamX Mk II imaging flow cytometer with × 60 magnification. Compensation was done using ultracomp beads (Invitrogen cat. # 01-2222-41) with the corresponding fluorophores. Image analysis was done using the Amnis IDEAS Software where cells were gated for live/dead using a dye and single-focused cells using the areas and gradient RMS separately. PBMCs were gated for CD14, CD3, and CD19.

### Surface-FISHseq analysis

#### Single-cell RNA sequencing and analysis

PBMCs were isolated either through FACS (BD FACSAria™ Fusion flow cytometer) or biotin pulldown for maxRNA positive and negative cells. We used Chromium Single Cell 3′ Reagent kit v3.1 (10x genomics) to generate single-cell RNA-seq libraries, which were sequenced using the Illumina HiSeq4000 (IGM Genomics Center, UCSD). Single-cell clustering and visualization were done using Seurat 3 [58], and the classification of cells was done using the singleCellNet R package [22]. The training set for singleCellNet analysis was generated using a published single-cell transcriptome dataset from 68 k PBMCs [59].

#### Antisense probe hybridization

Oligo probe libraries were denatured at 70 °C, 5 min and kept at 37 °C before hybridization. Cells or PBMCs were suspended in culture media at 1 million cells/mL and were incubated with an oligonucleotide library (final concentration 10 nM) for 1 h at 37 °C. Cells were washed three times in 1x PBS and pelleted by centrifuging at 500×*g* for 5 min.

#### Psoralen cross-linking

The cell pellet was resuspended (~ 250 million cells) in 5 mL of ice-cold 4′-Aminomethyltrioxsalen hydrochloride (AMT) solution (0.5 mg/mL in PBS; Sigma, cat. #A4330) and incubated on ice for 15 min. The cell suspension was transferred to a pre-chilled 10-cm tissue culture dish, which was then placed on ice under a long-wave UV bulb (350 nm) in a UV Stratalinker 2400 (Stratagene). Cells should be approximately 3–4 cm away from the light source. Crosslinking of probe-maxRNA chimeras was performed under UV light at maximum power (6500 μW/cm^2^) for 10 min, with gentle mixing every 2.5 min. Cells were then transferred to cold tubes and pelleted at 330×*g* for 4 min.

#### Membrane isolation

Cells were resuspended in 1 mL hypotonic lysis buffer (10 mM Tris-HCl pH = 7.5, 10 mM KCl, 2 mM MgCl2, 33 μL protease inhibitor cocktail (Sigma) and 10 μL RNasin per 10 mL of solution) and incubated on ice for 15 min. Cell membranes were disrupted using a 2 mL Dounce homogenizer with a tight-fitting pestle (Size B) for 20 passes. The homogenates were transferred into an ultracentrifuge tube and centrifuged at 20,000*g* for 20 min to remove membrane-bounded organelles. In a fresh ultracentrifuge tube, the supernatant was centrifuged again at 100,000×*g* for 35 min, to pellet the membrane and remove ribosomes, RNAs, and other cytosolic molecules. The pellet was resuspended in storage buffer (10 mM Tris-HCl pH 7.5, 1 mM EDTA, and 10 μL RNasin per 10 mL of solution). Centrifuge at 100,000×*g* for 35 min. Membrane-associated RNA concentration was quantified by Qubit.

#### maxRNA streptavidin bead pulldown

Streptavidin C1 (Invitrogen) beads were subjected to RNase removal treatment, blocked in BSA/PBST at 4 °C until ready. The beads were washed in B&W buffer (10 mM Tris-HCl [pH 7.5], 1 mM EDTA, 2 M NaCl) and incubated membrane extracts per at 4 °C for 1 h and RT for 30 min. Beads were washed three times in urea low salt wash buffer (8 M Urea, 10 mM Tris-HCl pH 7.5, 1 mM EDTA, 0.10% NP-40, 150 mM NaCl, 0.50% SDS), three times in high salt wash buffer (10 mM Tris-HCl pH 7.5, 4 M NaCl, 1 mM EDTA, 0.2% Tween 20), and once with PNK wash buffer (20 mM Tris-HCl pH 7.5, 10 mM MgCl2). Reverse cross-linking were performed in the plate under a short-wave UV bulb (265 nm) in a UV Stratalinker 2400 (Stratagene).

#### maxRNA library generation and sequencing

Libraries were generated using the NEB Low Input/Single Cell library prep kit (NEB cat. #E6420S). Paired-end sequencing of the library was performed using Illumina Miniseq Platform, using the High Output reagent kit (150-cycles).

#### Computational analysis of Surface-FISHseq

Adapter sequences and low-quality reads were trimmed from the raw sequence data with the Trimmomatic package [54]. A minimum of 50-bp read length filter was applied. Sequence alignment to the human genome (GRCh38/hg38) was performed using STAR (v2.7.3a) [55]. The resulting bam file was sorted and uniquely mapped reads were counted using featureCounts [56] with GENCODE human gene annotation (GRCh38, release 33).

The genes with the normalized counts ≤ 5 were excluded from further analysis. Two sample comparison of the test libraries vs. the control libraries was carried out by using the R/Bioconductor package DESeq2 [14] requiring the FDR (Benjamini-Hochberg adjustment) > 0.15 and fold change > 2.

Pathways analysis was performed using the Toppgene Suite [60]. Top non-redundant pathways were plotted for genes identified in two or more Surface-FISH-seq experiments. Pathways were filtered by FDR and ranked by the ratio between genes from input versus total genes in ontology, as indicated on the *y*-axis.

### Monocyte attachment assay

CD14+ monocytes were isolated directly from human whole blood samples using StraightFrom LRSC CD14 MicroBead Kit (Miltenyi Biotec, cat. #130-117-026), following the manufacturer’s protocol. Cells were stained with Calcein AM (2.5 μM, Biolegend cat. #425201) for 15 min at 37 °C for live-cell fluorescent tracking. Meanwhile, oligonucleotide probes were denatured at 70 °C, 5 min, and kept at 37 °C before hybridization. Cells were washed and resuspended in serum-free RPMI media at 10^6^ cells/mL and evenly segregated for hybridization with oligonucleotide probes at an equal molar concentration (10 nM) for 1 h at 37 °C.

After incubation, cells were washed thoroughly in PBS for three times and were seeded onto a confluent layer of HUVECs (RRID:CVCL_2959) in 96-well plates prepared 48–72 h before the experiment. Cells were incubated for 30 min to allow for attachment. Unattached cells were removed with PBS washes. Attachment of the fluorescently labeled monocytes was quantified using the SpectraMax i3x Multi-Mode Microplate Reader from 4 wells, with the sum value from 132 read points per well as final fluorescence output. Statistical summary and tests were performed in Prism 8. *P* values were generated by Kruskal-Wallis nonparametric test for multiple comparisons against the control groups with Bonferroni adjustments.

## Supplementary information

**Additional file 1: Fig. S1.** Size distribution of surface RNAs analyzed in Surface-seq. **Fig. S2.** Size distribution of the Surface-seq constructed sequencing libraries. **Fig. S3.** TTD microscopy of EL4 cells. **Fig. S4.** Images of healthy donor PBMCs from imaging flow cytometry. **Fig. S5.** tSNE plots of isFISH sorted single cells. **Fig. S6.** Summary of Surface-FISHseq datasets. **Table S1.** Summary of Surface-seq libraries. **Table S2.** Summary of Surface-FISHseq libraries. **Table S3.** Probe sequences for Surface-FISH.

## Data Availability

All the sequencing datasets generated in this study are available at NCBI’s Gene Expression Omnibus (GEO) with additional processed data files under accession number GSE150237 (https://www.ncbi.nlm.nih.gov/geo/query/acc.cgi?acc=GSE150237) [61].
