# Supplementary material for: Natural display of nuclear-encoded RNA on the cell surface and its impact on cell interaction
Source: Genome Biol. 2020 Sep 10;21:225. doi: 10.1186/s13059-020-02145-6 (PMC7488101; doi:10.1186/s13059-020-02145-6)
Supplement: Supplementary file 1 — Additional file 1: Fig. S1. Size distribution of surface RNAs analyzed in Surface-seq. Fig. S2. Size distribution of the Surface-seq constructed sequencing libraries. Fig. S3. TTD microscopy of EL4 cells. Fig. S4. Images of healthy donor PBMCs from imaging flow cytometry. Fig. S5. tSNE plots of isFISH sorted single cells. Fig. S6. Summary of Surface-FISHseq datasets. Table S1. Summary of Surface-seq libraries. Table S2. Summary of Surface-FISHseq libraries. Table S3. Probe sequences for Surface-FISH. [file 13059_2020_2145_MOESM1_ESM.pdf]

Supplementary figures

Fig. S1: Size distribution of surface RNAs analyzed in Surface-seq. (A) Bioanalyzer derived fluorescence intensity (y axis) versus RNA size (x axis) from EL4 surface RNAs extracted by Surface-seq variation 1. (B-C) Two biological replicates of (A). (D-E) Bioanalyzer derived fluorescence intensity (y axis) versus RNA size (x axis) from EL4 surface RNAs extracted by Surface-seq variation 2 from 2 biological replicates. (F) RNA ladder. FU: fluorescence intensity.

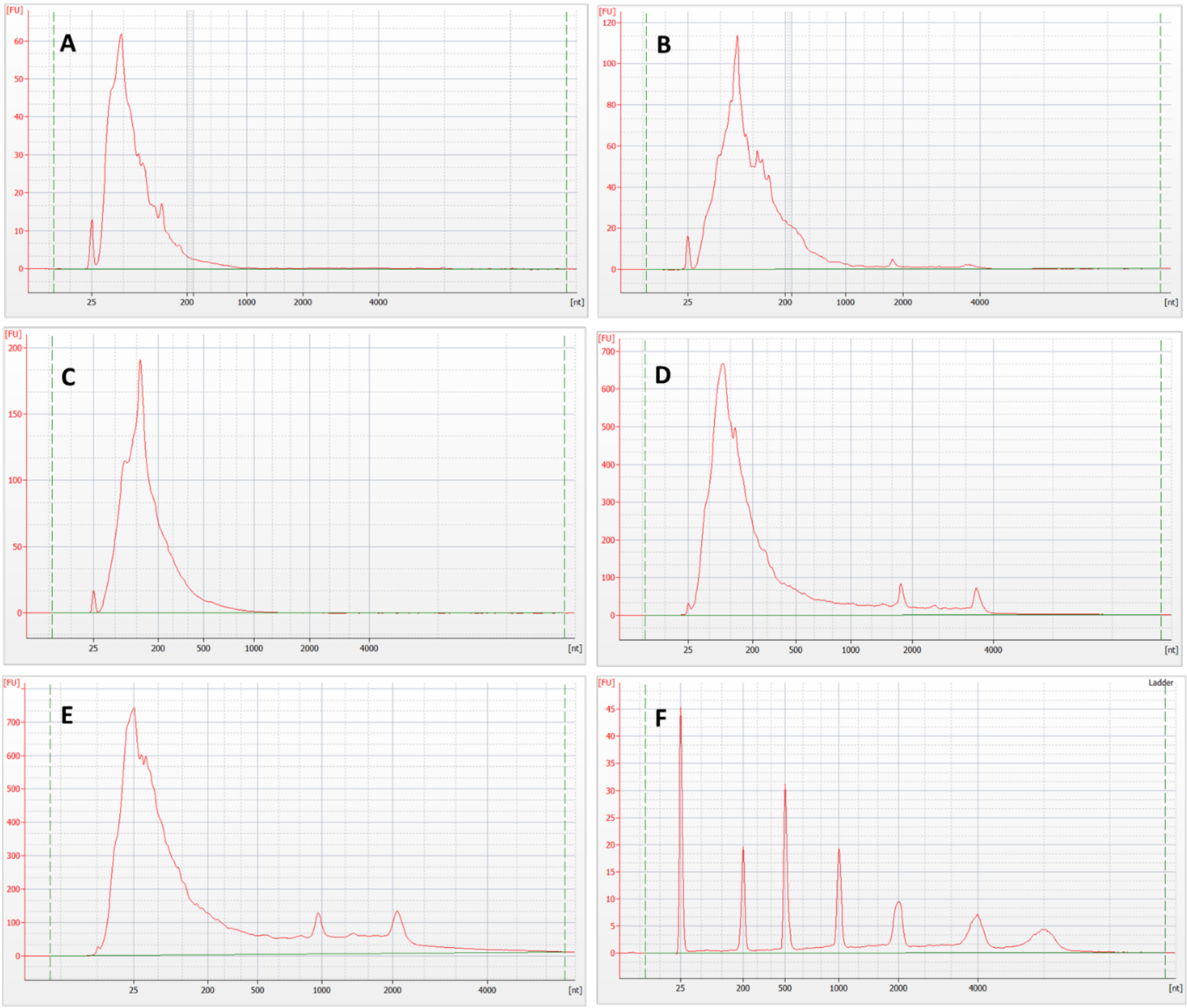

Fig. S2: Size distribution of the Surface-seq constructed sequencing libraries. (A-C) Fluorescence intensity (y axis) versus DNA size (x axis) from 3 biological replicates obtained by Surface-seq variation 1, measured by TapeStation (A, B) and bioanalyzer (C). (D-E) Fluorescence intensity (y axis) versus DNA size (x axis) from 2 biological replicates obtained by Surface-seq variation 2. (F) DNA ladder. FU: fluorescence intensity.

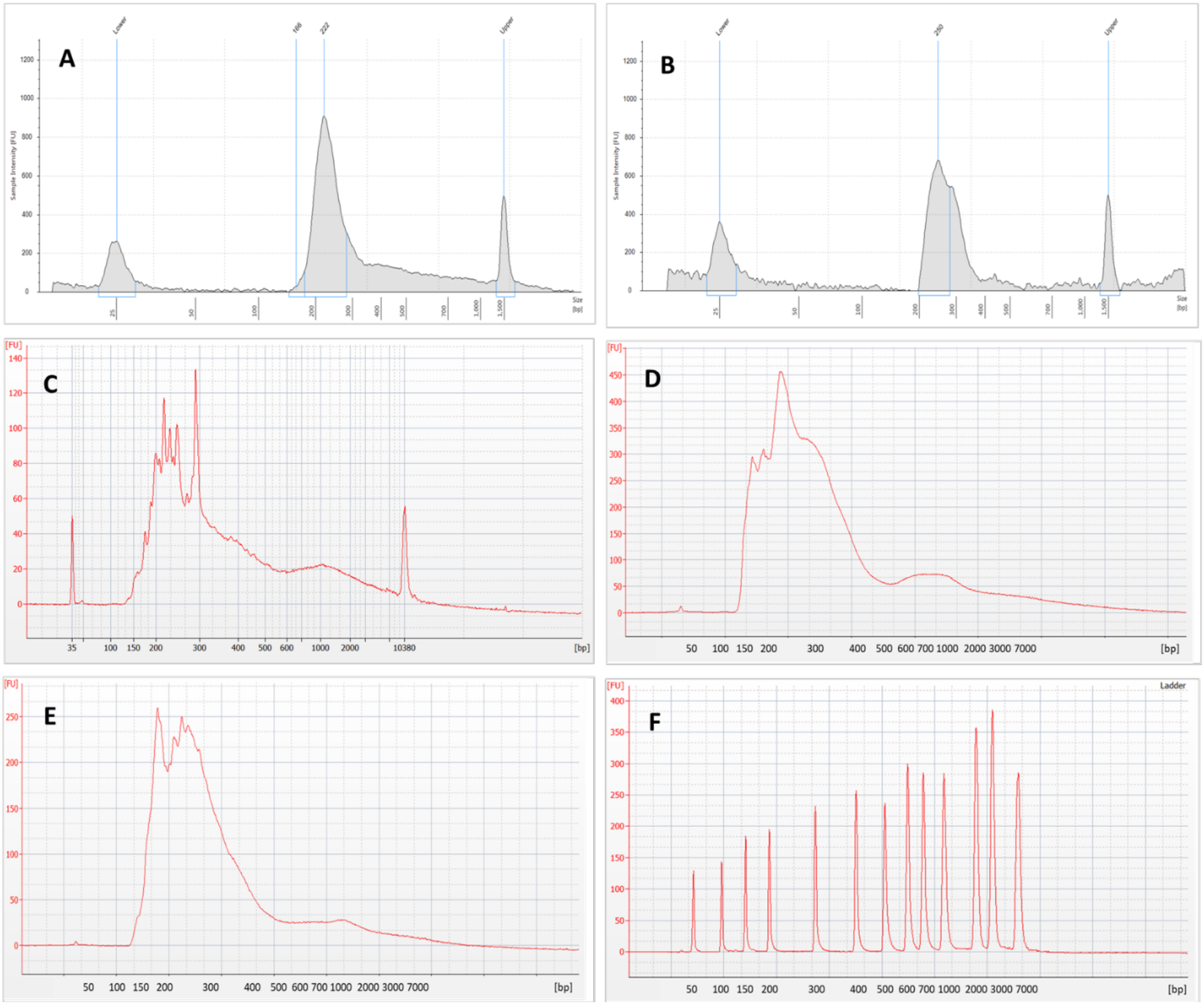

Fig. S3: TTD microscopy of EL4 cells. The membrane permeable dye (CellTracker Orange) and the membrane impermeant quencher acid blue 9 (AB9) were applied to the same cells. AB9 cannot enter the cell with intact membrane and thus cannot quench the membrane permeable dye, resulting in fluorescence signals in cells with intact membrane (green arrow). Leaky (orange arrow) or damaged membrane (red arrow) allows for the quencher to enter the cell, resulting in reduced or diminished fluorescence of the cell.

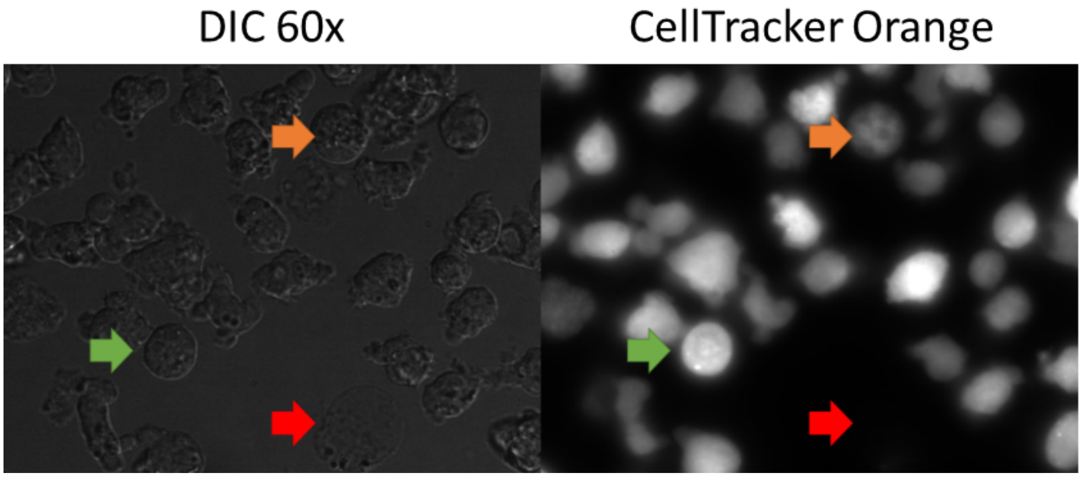

Fig. S4: Images of healthy donor PBMCs from imaging flow cytometry. Cells were labeled with cell display markers CD14, CD3ε, and CD19 to differentiate between monocyte, T cell and B cell populations, respectively. maxRNA was hybridized with antisense oligos and observed primarily on CD14 presenting cells.

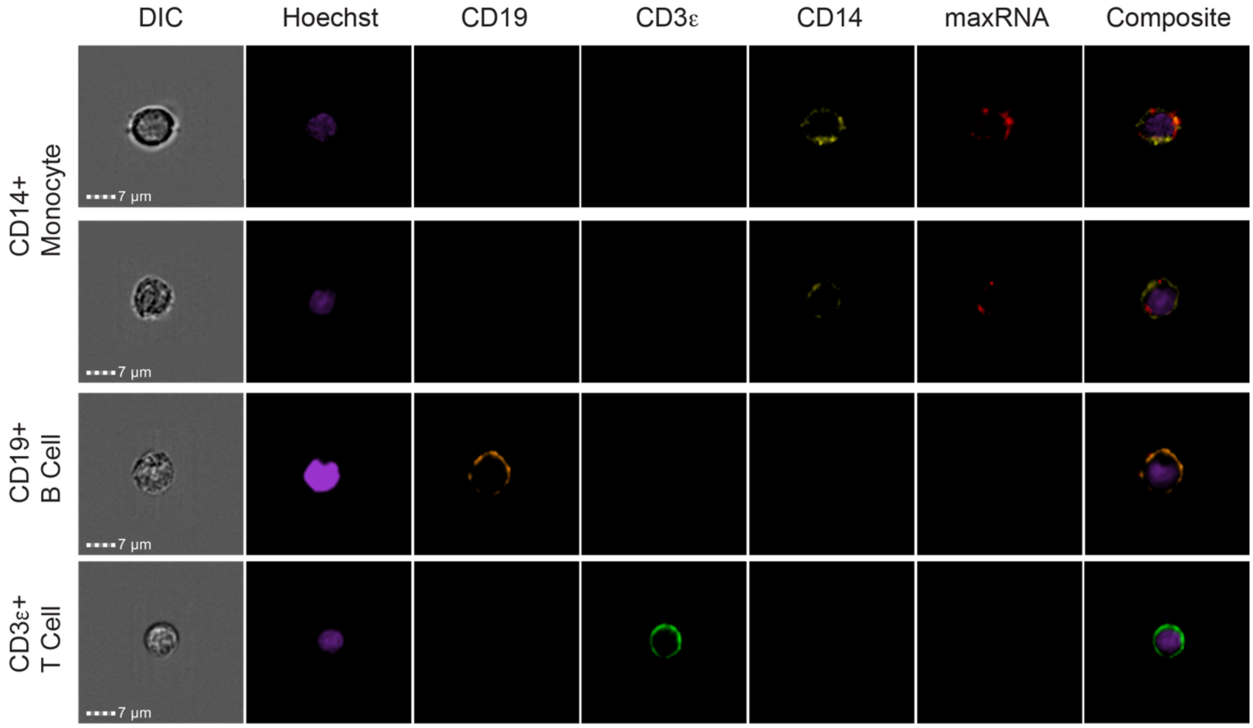

Fig. S5: tSNE plots of isFISH sorted single cells. isFISH+ (pink) and isFISH- (blue) cell clusters, and color-coded expression levels for T cell marker *CD3E*, *CD8A*, natural killer (NK) cell marker *NKG7*, and B cell marker *MS4A1*.

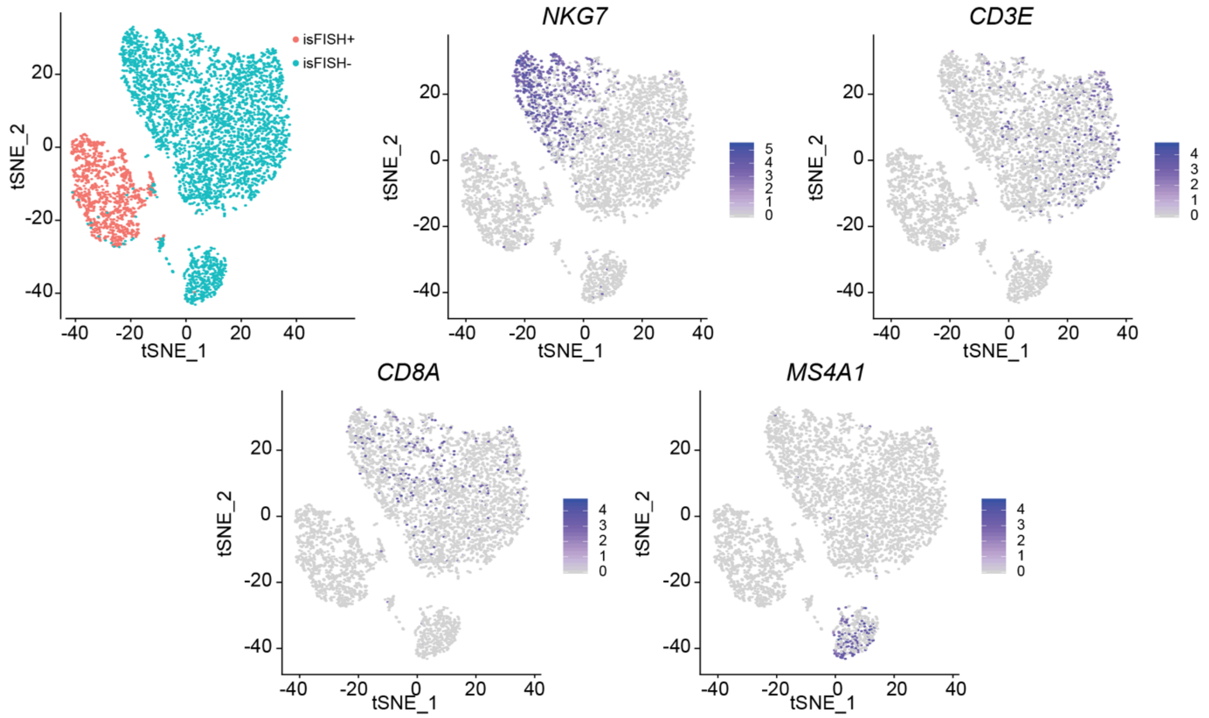

Fig. S6: Summary of Surface-FISHseq datasets. (A) The numbers of reads (blue) and uniquely mapped reads (UMR) for every Surface-FISHseq library (column), including 3 test (Membrane+) and control libraries (Membrane control) from the Surface-FISHseq-membrane experiment, the test (FACS+) and control libraries (FACS control) from the Surface-FISHseq-FACS experiment, and the test (Psoralen+) and control libraries (Psoralen control) from the Surface-FISHseq-Psoralen experiment. (B) The distribution of identified maxRNAs by transcript type. (C) The GO terms enriched in maxRNA genes (FDR < 10^{-10}), ranked by the ratio of the maxRNA genes vs. the total number of genes in this GO term (ratio of maxRNA genes in this GO term).

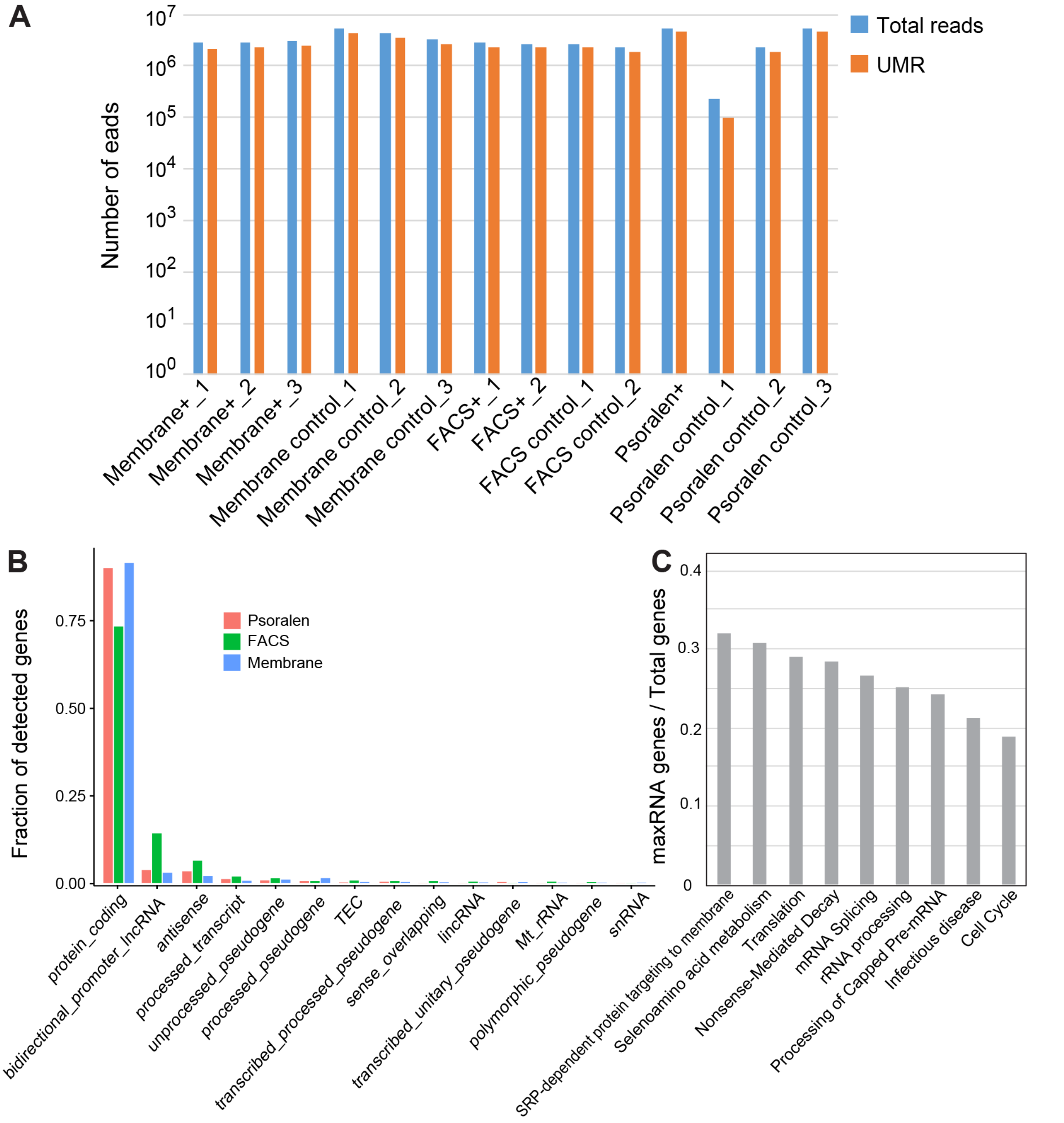

Supplementary tables

Table S1: Summary of Surface-seq libraries. The two Surface-seq technical variations are labeled as A and B. Input cells: the approximate number of input cells. Library concentration: the concentration of the final sequencing library. Total reads: the total number of sequencing reads. UMR: uniquely mapped reads

Sample ID	Technical variation	Input cells (million)	Concentration (nM)	Total reads	UMR
**A1**	A	400	24	22,117,239	9,734,312
**A2**	A	400	20	62,069,245	29,931,521
**A3**	A	400	44	15,694,988	8,038,032
**B1**	B	400	88.6	120,294,072	55,617,225
**B2**	B	400	36.6	106,187,703	56,243,455

Table S2: Summary of Surface-FISHseq libraries. UMR: number of uniquely mapped reads. UMP: percentage of uniquely mapped reads.

Technical variation	Experiment	Group	ID	Donor ID	Total Reads	UMR	UMP
**Psoralen**	Probe library pulldown	Test	PP_1	9799	5457193	4697546	86.08%
	Non-crosslinked library pulldown	Control	PN_C1	9799	234808	95467	40.66%
	Non-crosslinked dArt4 pulldown	Control	PN_C2	9799	5180356	4606128	88.92%
	Probe dArt4 pulldown	Control	PN_1	9799	2377320	1909222	80.31%
**FACS**	FACS+ pulldown	Test	FP_1	575	2759220	2301510	83.41%
	FACS+ pulldown	Test	FP_2	575	2723909	2327848	85.46%
	FACS- pulldown	Control	FN_1	561	2797693	2325819	83.13%
	FACS- pulldown	Control	FN_2	561	2253317	1865421	82.79%
**Membrane Purification**	Mem pulldown	Test	MP_1	561	2811824	2099391	74.66%
	Mem pulldown	Test	MP_2	575	2850086	2325989	81.61%
	Mem pulldown	Test	MP_3	730	3135792	2443210	77.91%
	Total membrane	Control	MT_1	561	5381193	4420311	82.14%
	Total membrane	Control	MT_2	575	4233565	3497718	82.62%
	Total membrane	Control	MT_3	730	3369136	2663567	79.06%

Table S3: Probe sequences for Surface-FISH. C30 represents a 30-carbon spacer and N bases refer to equal mixture of A, C, T, or G bases.

**Sequence Name**	**Sequence**
Neat1-1	Amine-C30-GAATGTCTGAGTTCTTGGCCAGCCTGGTTTACAAA
Neat1-2	Amine-C30-CAATCCAAGGCTTCTGGTTGAGGAATGGTGATAG
Neat1-3	Amine-C30-CACATTTGGGAGGCAGTCATTAGTAGATCTTTGAA
Neat1-4	Amine-C30-GTTTTCAGTTAAGAATCCCTCTGACCAATGCAG
Neat1-5	Amine-C30-CAAGGTTTTAAGTGACCCCTTAACCTCAGAGTGAG
Malat1-1	Amine-C30-GTTACAAATAAACACAAGTATACAATGCACAAGAAG
Malat1-2	Amine-C30-GCCACTTCCTTTGTTCCTATAGTAGTTATTAAGAT
Malat1-3	Amine-C30-CTTGATAATATAAAAGCTATCACCAGAAGAAATTCC
Malat1-4	Amine-C30-CTACAATCTATATTCATCCAACAGCTTCAGAAGAG
Malat1-5	Amine-C30-CTGAAATCATAAACTAAACAATTACCTAACACCCC
Malat1_Mut	Amine-C30-GTTACAAATAAACANNNNNNTACAATGCACAAGAA
Neat1_Mut	Amine-C30-GAATGTCTGAGTTCTTNNNNNNCCTGGTTTACAAA
